# The impact of study factors in the association of periodontal disease and cognitive disorders: systematic review and meta-analysis

**DOI:** 10.1093/ageing/afad015

**Published:** 2023-02-14

**Authors:** Harriet Larvin, Chenyi Gao, Jing Kang, Vishal R Aggarwal, Susan Pavitt, Jianhua Wu

**Affiliations:** School of Dentistry, University of Leeds, Leeds, UK; School of Dentistry, University of Leeds, Leeds, UK; Oral Biology, School of Dentistry, University of Leeds, Leeds, UK; School of Dentistry, University of Leeds, Leeds, UK; School of Dentistry, University of Leeds, Leeds, UK; School of Dentistry, University of Leeds, Leeds, UK; Centre for Primary Care, Wolfson Institute of Population Health, Queen Mary University of London, London, UK

**Keywords:** Periodontitis, dementia, cognitive decline, cohort studies, systematic review, older people

## Abstract

**Aim:**

The aim was to assess study factors that impact the association of cognitive disorders in people with periodontal disease (PD).

**Method:**

Medline, EMBASE and Cochrane databases were searched until February 2022 using keywords and MeSH: (periodon^*^ OR tooth loss OR missing teeth) AND (dementia OR Alzheimer’s Disease OR cognitive^*^). Observational studies reporting prevalence or risk of cognitive decline, dementia or Alzheimer’s disease (AD) in people with PD compared with healthy controls were included. Meta-analysis quantified the prevalence and risk (relative risk[RR]) of cognitive decline, dementia/AD, respectively. Meta-regression/subgroup analysis explored the impact of study factors including PD severity and classification type, and gender.

**Results:**

Overall, 39 studies were eligible for meta-analysis: 13 cross-sectional and 26 longitudinal studies. PD demonstrated increased risks of cognitive disorders (cognitive decline—RR = 1.33, 95% CI = 1.13–1.55; dementia/AD—RR = 1.22, 95% CI = 1.14–1.31). Risk of cognitive decline increased with PD severity (moderate—[RR] = 1.14, 95% confidence interval [CI] = 1.07–1.22; severe—RR = 1.25, 95% CI = 1.18–1.32). For every 10% population increase in females, the risk of cognitive decline increased by 34% (RR = 1.34, 95% CI = 1.16–1.55). Self-reported PD showed a lower risk of cognitive disorders compared with clinical classification (cognitive decline—RR = 0.77, 95% CI = 0.65–0.91; dementia/AD—RR = 0.86, 95% CI = 0.77–0.96).

**Conclusion:**

The prevalence and risk estimates of cognitive disorders in association with PD can be influenced by gender, the disease classification of PD and its severity. Further homologous evidence taking these study factors into consideration is needed to form robust conclusions.

## Key Points

- A systematic review that explores study factors impacting the association of cognitive disorders in periodontal disease (PD).
- Study factors including PD severity, classification and sex influence prevalence and risk estimates of cognitive disorders.
- Further homologous evidence from observational studies is needed to form robust conclusions.

## Introduction

Periodontal disease (PD), a chronic inflammatory condition, is a major driver of tooth loss in older age and the sixth most prevalent non-communicable disease worldwide [1]. Dementia is the fifth leading cause of death globally and there are concerns that the disease prevalence could increase at an alarming rate as a result of the ageing population [2]. Observational evidence suggests that cognitive decline, as a pre-cursor to dementia, is associated with fewer teeth [3–6]. Recent evidence also suggests that there may be a reciprocal relationship between poor oral health and dementia [7]. Experimental studies have shown chronic systemic inflammation may be linked to onset of both dementia and PD [8, 9], and there is also evidence of increased levels of inflammatory markers associated with periodontal pathogens in people with Alzheimer’s disease (AD) [10]. Understanding the factors that could influence this association is imperative in view of tailoring public health initiatives promoting oral health towards dementia prevention.

Observational studies enable non-intrusive examination of exposures, outcomes and risk factors in the general population. Previous systematic reviews have sought to quantify the prevalence and risk of cognitive disorders in PD using observational studies; however, meta-analyses of effect sizes vary vastly and often conclude that further evidence is required to substantiate the findings [11–14]. One review suggested that combining cross-sectional and longitudinal studies in a meta-analysis caused around 16% of heterogeneity [12]. Given their real-world setting, observational studies can be subject to several biases including confounding and selection; it is therefore crucial to consider study factors when conducting systematic reviews and meta-analyses accordingly [15]. Furthermore, recent work has suggested there is risk of overestimating the link of PD and cognitive disorders from spurious associations identified in cross-sectional research [16].

We previously demonstrated the utility of meta-regression in revealing the effect of study factors such as sex, PD classification and study region on risk estimates of cardiovascular disease [17]. A recent systematic review used similar methods to explore effect of sample size, treatment during the follow-up and bias rating in studies estimating the risk of adverse pregnancy outcomes and diabetes in people with PD [18]. As yet, no study has examined the effects of study characteristics on prevalence and risk estimates of cognitive disorders, specifically cognitive decline/impairment and dementia, in people with PD. The aim of the current investigation was to assess the study factors that could impact the association of cognitive disorders in people with PD. In order to pool the results of individual studies, a meta-analysis will be used to quantify risk of dementia in PD populations and meta-regression will be used to evaluate the impact of key risk factors.

## Methods

Study design—a systematic review of cross-sectional and longitudinal cohort studies that examine the prevalence and incidence of cognitive disorders in people with periodontitis.

### Search strategy and selection criteria

The search string considered alternate terms incorporating several relevant key words and Medical Subject Headings (MeSH) headings. The final Boolean search string was: (periodon^*^ OR tooth loss OR missing teeth) AND (dementia OR Alzheimer’s Disease OR cognitive^*^) (Supplementary Table 1). The search string was applied from database conception until 2 February 2022 to Medline, EMBASE and Cochrane databases to ensure retrieval of a broad scope of literature. Additional reference checking and ‘citation snowballing’ methods of key articles were also undertaken to maximise search sensitivity.

Study inclusion criteria were outlined as the following:

- Cross-sectional or longitudinal retrospective/prospective cohort.
- Clinically diagnosed or self-reported PD.
- Clearly defined classification of dementia, AD and/or subtypes such as vascular dementia, or cognitive decline (including mild cognitive impairment). Diagnosis should be identified via appropriate disease classification codes such as ICD-10F00-F03, or clinical assessment using verified assessment tool such as MMSE or MoCA.
- Provides estimates for prevalence (cross-sectional) or incidence (longitudinal) of dementia or cognitive decline, and/or when absent raw numbers are available for crude calculation.
- Peer reviewed articles and published in English.

For full details of study selection, see Note S1 in the supplemental file.

### Quality assessment

Quality assessment tools for observational studies can be contentious [19]; therefore, this review employed the Risk of Bias in Non-Randomised Studies of Interventions (ROBINS-I) recommended by Cochrane to determine the risk of bias in cohort and longitudinal observational studies [20]. Results from the risk of bias assessment were conferred with a second author and discrepancies discussed before finalising ROBINS-I assessment table.

The protocol for the present review was registered to PROSPERO before the study began (registration number: CRD42019154897).

### Statistical analysis

Odds ratios (OR), hazard ratios (HR) and relative risks (RR) were used in different studies to quantify the risk of cognitive decline and dementia/AD. We examined cross-sectional and longitudinal studies separately. In cross-sectional studies that did not report the effect size, we used raw numbers of exposed/unexposed and case numbers to quantify a crude RR in pooling for meta-analysis. We converted ORs and HRs into RR in order to maximise the number of included studies for meta-analysis [21]. Where possible, adjusted RRs were used in the meta-analysis and adjustments of key confounders, such as smoking, gender and age, were screened for each study. For inclusion in meta-analysis, studies must have reported total population numbers for PD and non-PD cases, and RRs or converted RRs should also be available to be eligible for synthesis and pooling. For precision, studies should also have a minimum of 30 participants in the exposed PD/unexposed groups; studies that reported less than this were included as part of a sensitivity analysis.

Random effects meta-analysis was performed for prevalence or risk of cognitive decline or dementia according to the study type (cross-sectional or longitudinal). Subgroup analysis and meta-regression examined the impact of study and population factors such as age, smoking, PD classification (self-report or clinical), study region, sex and sample size. Study and population factors were selected according to previous literature and data availability. Average age (mean or median), smoking (population percentage), sex (female population percentage) were treated as continuous variables in meta-regression. Where age was reported in bands and the average was missing, the median value of the mode group was considered the average. PD classification, study region and sample size were treated as categorical variables. Sample size categories were determined according to the range in sample sizes within included studies and to maximise study numbers. Variables included in the meta-regression were dependent on data availability from included studies. *I*^2^ was used to measure the study heterogeneity. Publication bias was illustrated with funnel plots, which was quantified by Egger’s test. Forest plots were used to visualise the pooled results from meta-analysis.

## Results

The search strategy retrieved 2,146 studies, with 1,726 studies eligible for title and abstract screening following duplicate removal. After title and abstract screening and hand searches, 232 studies were eligible for full text screening with 49 studies eligible for review. Most studies were excluded due to ineligible study design (*n* = 63). Of the 49 included studies, 21 were cross-sectional and 28 were of longitudinal design, including 11 and 11, and 20 and 9 studies examining dementia or cognitive decline, respectively. Two studies examined both dementia and cognitive decline as outcomes [22, 23]. One study was not eligible for meta-analysis due to missing raw data [24] and a further study was not eligible due to insufficient case numbers (number of cases in exposed = 0) [25]. One study was not eligible for meta-regression due to the across-region study population [22]. Seven studies were not eligible for meta-analysis as they reported below 30 participants in the exposed/unexposed groups [23, 26–31]. All included studies were published between 2007 and 2022 (Figure S1, Table 1).

**Table 1:** Summary of included studies

	**Study**	**Region**	**Age (average)**	**Females (%)**	**Sample size**	**PD classification**	**Total follow-up time (years)**	**ROBINS-I rating**
***Cross-sectional studies***								
Laugisch 2021^*^^*^^*^	AD	Europe	55	58.3	40	Clinical		Serious
Popovac 2021^*^^*^^*^	AD	Europe	62.6	76.06	179	Clinical		Serious
Tiisanoja 2019	AD	Asia	70	80.9	170	Clinical		Moderate
Tsuneishi 2021	AD	Asia	55.2	66.5	3,549,513	Clinical		Serious
Okamoto 2010	Cognitive decline	Asia	49.6	71	1964	Clinical		Serious
Winning 2022	Cognitive decline	Europe	55.3	65.5	2,258	Clinical		Moderate
Abdulhade Ganem 2019	Cognitive decline	Asia	100	48.2	79	Clinical		Critical
ALFotawi 2019	Cognitive decline	Asia	40	65.67	68	Clinical		Serious
Jockusch 2021^*^^*^^*^	Cognitive decline	North America	65.7	86	25	Clinical		Serious
Kim 2021	Cognitive decline	Asia	65.7	77.2	134	Clinical		Serious
Mizutani 2021 ^*^^*^	Cognitive decline	Asia	70	71	35	Clinical		Serious
Nilsson 2014	Cognitive decline	Europe	58.2	88.5	942	Clinical		Serious
Nilsson 2018	Cognitive decline	Europe	55	88.5	767	Clinical		Serious
Peres 2014	Cognitive decline	South America	62.5	62.5	1,122	Self-report		Moderate
Sharma 2021^*^^*^^*^	Cognitive decline	Asia	46.9	68.27	57	Clinical		Serious
Shin 2016	Cognitive decline	Asia	48.1	69.2	108	Clinical		Serious
Barbe 2019^*^^*^^*^	Dementia	Europe	73	82	40	Clinical		Serious
Chu 2015^*^^*^^*^	Dementia	Asia	79.7	80	97	Clinical		Serious
Gao 2020	Dementia	Asia	79	80.9	167	Clinical		Serious
Kato 2019	Dementia	Asia	51	78.1	210	Clinical		Serious
Saito 2021	Dementia	Asia	56.6	78	3,108	Clinical		Moderate
***Longitudinal studies***								
Adam 2022	AD	North America	58	76.5	162	Clinical	17	Serious
Chen 2017	AD	Asia	47	54.2	27,963	Clinical	7	Serious
Batty 2013	Cognitive decline	Asia, Australasia, Europe and North America	42.5	65.4	8,788	Self-report	5	Serious
Hatta 2018	Cognitive decline	Asia	52.9	80	463	Clinical	3	Serious
Nilsson 2018	Cognitive decline	Europe	56.4	67	566	Clinical	6	Serious
Govindan 2021	Cognitive decline	Asia	69.3	72.5	120	Clinical	5	Serious
Iwasaki 2019	Cognitive decline	Asia	52.4	80.1	179	Clinical	5	Moderate
Okamoto 2015	Cognitive decline	Asia	49.4	71	2,155	Clinical	5	Critical
Saito 2018	Cognitive decline	Asia	70.3	72.05	140	Clinical	11	Serious
Xu 2021	Cognitive decline	Asia	49	81.4	6,721	Self-report	8	Serious
Yang 2022	Cognitive decline	Asia	52.7	83	7,098	Self-report	16	Serious
Arrive 2012 ^*^^*^	Dementia	Europe	54.6	70	405	Clinical	15	Critical
Choi 2019^*^	Dementia	Asia	49.3	60.4	262,349	Clinical	12	Serious
Demmer 2020	Dementia	North America	54.4	63	3,258	Clinical	18.4	Moderate
Holmer 2022	Dementia	Europe	43.8	61	37,174	Clinical	8	Critical
Kiuchi 2021	Dementia	Asia	54	73.1	35,744	Self-report	6	Serious
Lee 2017a^*^	Dementia	Asia	50.5	54.5	117,476	Clinical	10	Serious
Lee 2017b^*^	Dementia	Asia	46	72.4	6,056	Clinical	12	Serious
Lee 2020^*^	Dementia	Asia	51.6	52	54,234	Clinical	13	Serious
Malone 2021^*^	Dementia	North America	38.4	67	439,760	Clinical	3	Serious
Paganini-Hill 2012	Dementia	North America	69	81	1,169	Self-report	18	Serious
Stein 2007^*^^*^^*^	Dementia	North America	100	84	101	Clinical	12	Serious
Stewart 2015	Dementia	Europe	100	80	351	Clinical	38	Serious
Takeuchi 2017	Dementia	Asia	55.7	75	1,241	Clinical	5	Serious
Tzeng 2016^*^	Dementia	Asia	61.4	44.5	8,828	Clinical	10	Serious
Yamamoto 2012	Dementia	Asia	51.2	67	2,919	Self-report	4	Serious
Yoo 2019^*^	Dementia	Asia	66.5	64.5	209,806	Clinical	14	Serious
Kim 2020^*^	Vascular dementia	Asia	28.5	44.5	9,807	Clinical	14	Serious

Key: Alzheimer’s disease, AD; periodontal disease, PD. ^*^Crude rate only, ^*^^*^received treatment during follow-up, ^*^^*^^*^not eligible for meta-analysis

Most study populations were from Asia (*n* = 18) and utilised a clinical diagnosis of PD to define the exposure (*n* = 22). Participants in eight dementia studies also received periodontal treatment during the follow-up as part of the study design. The median total study follow-up time for longitudinal studies was 10 years (interquartile range[IQR] = 5–14 years) (Table 1). Risk of bias assessment by ROBINS-I checklist demonstrated most included studies were of serious risk of bias due to risk of confounding or selection biases (*n* = 39; Table 2). Both cross-sectional and longitudinal cognitive decline studies had significant risk of publication bias, whereas this was not observed in dementia/AD studies (Figures S2 and S3).

**Table 2:** Meta-regression of cross-sectional studies demonstrating change in the prevalence of cognitive disorders in people with periodontal disease by a unit change in study design factors

	**Prevalence risk ratio (95% CI)**	
	**Cognitive decline**	**Dementia and AD**
*PD severity*		
Moderate	Ref	Ref
Severe	1.20 (0.78–1.85)	2.27 (0.93–5.53)
*Region*		
Europe	Ref	–
Asia	0.75 (0.49–1.17)	–
*Rate*		
Crude	Ref	Ref
Adjusted	1.24 (0.81–1.89)	1.83 (1.24–2.70)^*^^*^
*Sample size*		
< 1,000	Ref	Ref
≥ 1,000	0.92 (0.55–1.52)	1.31 (0.65–2.65)
*Bias rating*		
Moderate	Ref	Ref
Serious	1.10 (0.72–1.70)	0.67 (0.36–1.24)
*Other factors*		
Females (for every 10% population increase)	0.94 (0.86–1.03)	1.03 (0.70–1.52)
Average age (for every 10-year increase)	1.20 (1.11–1.29)^*^^*^^*^	0.95 (0.52–1.74)
Smoker (%)†	1.00 (0.93–1.09)	1.01 (0.92–1.10)

Key: Alzheimer’s disease, AD; periodontal disease, PD. −Insufficient number of studies. †Separate model including only studies that reported smoking rate. Significant at ^*^0.05, ^*^^*^0.01, ^*^^*^^*^0.001 levels

### Cognitive decline

**Figure 1:**
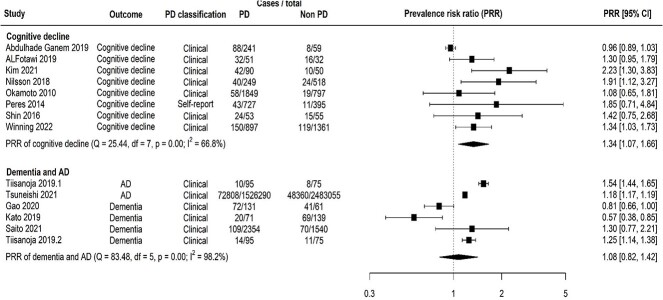
Forest plot showing results from random effect meta-analysis for the prevalence of cognitive disorders. Key: Alzheimer’s disease, AD; degrees of freedom, df; periodontal disease, PD; prevalence risk ratio, PRR.

**Figure 2:**
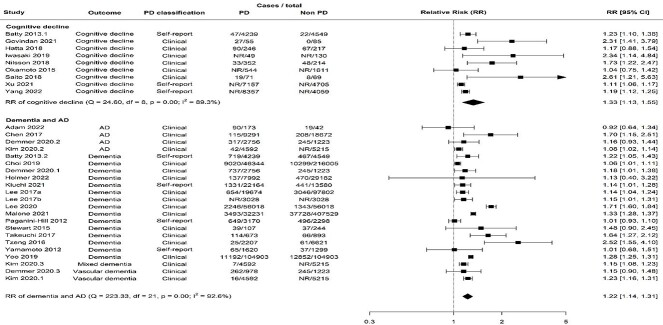
Forest plot showing results from random effect meta-analysis for the incident risk of cognitive disorders. Key: Alzheimer’s disease, AD; degrees of freedom, df; case numbers not reported, NR; periodontal disease, PD; relative risk, RR.

Random effects meta-analysis of cross-sectional studies showed that the prevalence of cognitive decline in people with PD was increased by 34% compared with those without PD (prevalence risk ratio [PRR] = 1.43, 95% confidence interval [CI] = 1.07–1.66; Figure 1). This outcome had moderate-high heterogeneity (${I}^2$ = 66.8%; Figure 1). The risk of developing cognitive decline in longitudinal studies was 33% higher in people with PD than those without (relative risk [RR] = 1.33, 95% CI = 1.13–1.55). The heterogeneity was high for longitudinal studies with this outcome (${I}^2$ = 89.3%; Figure 2).

Of note, a small study (*n* = 35) that was not eligible for meta-analysis identified no cases of cognitive decline in people without PD in non-smoking older Japanese outpatients [25].

### Cognitive decline—study factors

Subgroup analysis of cross-sectional studies revealed an incremental increase in prevalence of cognitive decline by PD severity and a reduction in heterogeneity (moderate—PRR = 1.21, 95% CI = 0.85–1.72, ${I}^2$ = 69.9%; severe—PRR = 1.35, 95% CI = 1.03–1.71, ${I}^2$ = 0%; Figure S4). The prevalence of cognitive decline was 20% higher in severe cases compared with moderate PD (PRR = 1.20, 95% CI = 0.78–1.85; Table 2). Prevalence estimates for cognitive decline were also impacted by study population (Figure S4) but meta-regression indicates this is not significant (Asia—PRR = 0.75, 95% CI = 0.49–1.17; Table 2). Older participants with PD had higher prevalence of cognitive decline compared with younger populations (younger—PRR = 1.17, 95% CI = 0.97–1.42; older—PRR = 2.06, 95% CI = 1.341–3.02; Figure S6). Meta-regression showed that for every 10 years increase in average age, there was a 20% increase in prevalence cognitive decline (PRR = 1.20, 95% CI = 1.11–1.29; Table 2).

For longitudinal studies, an incremental increase in the risk of cognitive decline by PD severity was also observed (moderate—RR = 1.14, 95% CI = 1.07–1.22; severe—RR = 1.25, 95% CI = 1.18–1.32; Figure S7). In fact, the risk of cognitive decline was 8% higher for those with severe PD compared with moderate cases (RR = 1.08, 95% CI = 0.84–1.38; Table 3). Furthermore, for every 10% population increase in females in the study population, there was a 34% increased risk of cognitive decline (RR = 1.34, 95% CI = 1.16–1.55; Table 3). The risk of cognitive decline in studies stratified by age was similar (younger—RR = 1.40, 95% CI = 1.01–1.94; older—RR = 1.36, 95% CI = 1.08–1.71; Figure S8). Compared with studies of moderate risk of bias, those of serious and critical risk reported 57 and 66% lower risks, respectively (serious—RR = 0.53, 95% CI = 0.31–0.92; critical—RR = 0.44, 95% CI = 0.24–0.82; Table 3). Meta-regression also showed that studies that utilised self-reported PD diagnosis reported 23% lower risks compared with clinical diagnosis (RR = 0.77, 95% CI = 0.65–0.91) and those of bigger sample sizes reported lower risks compared with sample sizes of less than 1,000 participants (1,000–10,000—RR = 0.65, 95% CI = 0.54–0.79; 10,000–100,000—RR = 0.66, 95% CI =  0.53–0.82; Table 3).

### Dementia and AD

Random effects meta-analysis of cross-sectional studies showed that the overall prevalence of dementia/AD was 8% higher in people with PD (PRR = 1.08, 95% CI = 0.82–1.42) with high heterogeneity (${I}^2$ = 98.2%; Figure 1). The incident risk of dementia/AD was also increased in people with periodontal disease in longitudinal studies (RR = 1.22, 95% CI = 1.14–1.31, ${I}^2$ = 92.6%; Figure 2).

### Dementia and AD—study factors

Incremental increase in prevalence of dementia/AD was observed in moderate PD (PRR = 0.98, 95% CI = 0.76–1.26) to severe cases (1.44, 95% CI = 0.89–2.32) when compared with those without PD (Figure S5). In fact, the prevalence of dementia and AD in severe PD was over 2-fold higher than those with moderate cases (PRR = 2.27, 95% CI = 0.93–5.53; Table 2).

PD severity did not appear to have impact on incident risk estimates of dementia and AD for longitudinal studies (Figure S7), with risks in moderate and severe PD similarly increased compared with mild cases (moderate—RR = 1.05, 95% CI = 0.81–1.37; severe—RR = 1.03, 95% CI = 0.78–1.35; Table 3). When compared with clinical PD diagnosis, self-reported PD showed reduced risks of dementia and AD (RR = 0.86, 95% CI = 0.77–0.96; Table 3). Risk of dementia and AD appeared highest in populations from Europe (RR = 1.41, 95% CI = 0.89–2.22; Figure S8). Meta-regression revealed lower risks of dementia and AD in studies from Asia and North America compared with those from Europe (Asia—RR = 0.87, 95% CI = 0.60–1.26; North America—RR = 0.77, 95% CI = 0.53–1.12; Table 3).

### Sensitivity analysis

Meta-analysis of longitudinal studies that reported periodontal treatment during the follow-up (*n* = 8) revealed an increased risk of dementia in people with PD of a similar magnitude to the main analysis (RR = 1.30, 95% CI = 1.14–1.48; Figure S10). Meta-regression revealed a modest 6% increase in risk of dementia and AD compared with studies that did not report periodontal treatment (RR = 1.06, 95% CI = 0.95–1.17; Table 3). Furthermore, including studies with fewer than 30 participants within exposed/unexposed groups did not greatly impact results of the meta-analysis of prevalence and risk of cognitive disorders in cross-sectional or longitudinal studies (Figures S11–S12).

## Discussion

In this systematic review, we examined 21 cross-sectional and 28 longitudinal studies reporting either prevalence or risk of cognitive decline, or dementia/AD. Overall, the prevalence and risk of cognitive decline was higher than dementia and AD in people with PD. Severe PD was associated with increased prevalence and risk of cognitive disorders. Meta-regression of study factors suggested that PD classification type, gender, age, study region and overall risk of bias may also attribute to variation observed in effect size estimates of observational studies.

**Table 3:** Meta-regression of longitudinal studies demonstrating change in the incident risk of cognitive disorders in people with PD by a unit change in study design factors

	**Relative risk (95% CI)**	
	**Cognitive decline**	**Dementia and AD**
*PD severity*		
Mild	–	Ref
Moderate	Ref	1.01 (0.77–1.34)
Severe	1.08 (0.84–1.38)	1.04 (0.79–1.38)
*Sample size*		
< 1,000	Ref	Ref
1,000–10,000	0.65 (0.54–0.79)^*^^*^^*^	1.06 (0.83–1.36)
10,000–100,000	0.66 (0.53–0.82)^*^^*^^*^	1.09 (0.82–1.46)
≥ 100,000	–	1.17 (0.90–1.52)
*PD classification*		
Clinical	Ref	Ref
Self-report	0.77 (0.65–0.91)^*^^*^	0.86 (0.77–0.96)^*^
*Region*		
Europe	–	Ref
Asia	–	0.87 (0.60–1.26)
North America	–	0.77 (0.53–1.12)
*Bias rating*		
Moderate	–	Ref
Serious	0.53 (0.31–0.92) ^*^	1.03 (0.89–1.20)
Critical	0.44 (0.24–0.82) ^*^^*^	–
*PD treatment received*		
Unknown	–	Ref
Yes	–	0.95 (0.85–1.05)
*Other factors*		
Females (for every 10% population increase)	1.34 (1.16–1.55)^*^^*^^*^	1.00 (0.96–1.04)
Average age (for every 10 year increase)	0.87 (0.77–1.00)	0.97 (0.93–1.01)
Smoker (%)	1.00 (0.99–1.01)	1.01 (1.00–1.01)^*^^*^
Total follow-up (for every 5 years)	0.94 (0.84–1.05)	0.96 (0.92–1.01)

Key: Alzheimer’s disease, AD; periodontal disease, PD. −Insufficient number of studies.

†Separate model including only studies that reported smoking rate. Significant at ^*^0.05, ^*^^*^0.01, ^*^^*^^*^0.001 levels

The findings of this review align with previous systematic reviews that have found augmented risks for cognitive decline and dementia/AD in people with PD [11–14]. Contrariwise, a recent review concluded that the evidence regarding periodontal pathogens and AD onset is contentious and subject to bias, which may influence the robustness of previous findings [32]. Evidence shows that age is a risk factor for PD and both cognitive decline and dementia/AD, with cognitive decline typically developing prior to a formal diagnosis of dementia/AD [33, 34]. We found that the prevalence and risks for cognitive decline were higher than for dementia/AD. This supports the notion that signs of cognitive decline are the early markers for subsequent neuro-degeneration and eventual dementia-onset [35]; thus, cognitive decline is a more frequently diagnosed condition than dementia [36]. There may also be differences in the association of dementia with other disease subtypes. For example, the risk of vascular dementia increases 2-fold in people diagnosed with cardiovascular disease [37]. PD is linked to augmented risks cardiovascular disease development [16], which could implicate vascular dementia development further along the disease trajectory. Further primary work is required to dissect the association of PD with specific subtypes of dementia.

Meta-regression has shown merit in exploring the impact of study factors on estimates for the risk of systemic diseases. Meta-regression and subgroup analyses by key study factors in the present review demonstrated reductions to statistical heterogeneity. We previously demonstrated using meta-regression that PD severity and male gender may increase estimates for risk of cardiovascular disease [17]. The former finding aligns with the current systematic review as we revealed that PD severity is incrementally associated with the prevalence of cognitive decline. We also showed that a higher proportion of females was associated with increased risks of cognitive disorders, though this could be reflective of the higher proportion of females with dementia than males [38]. People with self-reported PD had reportedly lower risks of cognitive disorders than those with clinical classification. This contrasts previous work that suggests classification of PD has no effect on longitudinal risk of cardiovascular disease [16]. A possible explanation could be the differences in severity of self-reported responses. For example, previous oral health research in the UK Biobank has utilised responses of bleeding gums (mild periodontitis/gingivitis) to loose teeth (indicative of severe periodontitis) [39, 40]. It is possible that studies that utilise a self-reported classification such as bleeding gums, a noticeable sign of disease, may have a higher proportion with mild/moderate PD, which may have a lower risk of developing cognitive disorders. These studies are also at risk of reporting bias and therefore the results may not be precise; however, there is evidence that suggests self-reported tools for PD are accurate [41]. A recent systematic review with meta-regression also revealed the sample size and risk of bias can impact study estimates for risks of adverse pregnancy outcomes and diabetes [18]. We found that sample size had a variable effect on estimates for cognitive disorders, whereas studies at serious risk of bias also did not affect the association of PD on dementia/AD compared with those of moderate risk. Generally, studies rated at moderate risk of bias used methods such as inverse probability weighting to account for selection biases and stratified random sampling [42–44]. Most studies were rates at serious risk of bias due to failing to address confounding and selection biases, thereby meta-regression of this factor may be problematic; as such, there is a need for better quality primary research in the field and researchers should interpret findings of systematic reviews with caution.

This systematic review with meta-analysis is the first of its kind to assess using meta-regression, the impact of study factors on effect size estimates for dementia and AD in PD; as such, the study has notable strengths. Through including both cross-sectional and longitudinal studies, as well as two systemic disease outcomes—cognitive decline and dementia/AD, we were able to examine the associations with PD using a larger pool of included studies. The use of meta-regression enabled adjustments for several key factors of study design including gender, PD severity, study region, age, risk of bias and sample size. This ensured a thorough exploration of effect sizes in association studies of PD and cognitive disorders. Our review is further strengthened through adherence to the PRISMA guidelines [45].

Although the primary aim of this review was to explore the causes of methodological heterogeneity through meta-regression, a limitation was the risk of bias present in the included studies due to selection and unmeasured cofounding. Given that the studies included in this review were cross-sectional and longitudinal design, often using real-world datasets such as electronic health records, this leaves opportunity for residual bias and statistical heterogeneity that cannot be adjusted for post-hoc. Furthermore, the results of meta-regression are dependent on sufficient sample size and we were not able to explore the influence of some study factors due to the absence of information in certain studies. The impact of subgroups demonstrated reductions in statistical heterogeneity, thereby advocating future homologous studies with transparent reporting to account for between-study variation. Furthermore, although we strove to account for classification bias through stratifying PD classification into self-reported versus clinical, the classification guidelines of both PD and cognitive disorders can change over time. Thus, true identification of these conditions are therefore dependant on the classification system used and the time of the study. Another limitation of this review is that we were not able to extract adjusted estimates of prevalence from all cross-sectional studies. As a result, these studies were at serious risk of confounding. Evidence suggests that PD is associated with multimorbidity [40, 46]; multimorbidity is also linked to worse outcomes in older age, including dementia incidence [47]. We were not able to explore effect of co-morbidities, and future work should account for multimorbidity and seek to make necessary adjustments. Other confounding factors such as deprivation and socioeconomic status should also be explored further in future meta-regression studies as known drivers of adverse health outcomes that may influence effect sizes.

This study demonstrates the fragility of estimations of the association between PD and cognitive disorders, with study factors such as age, gender, study region and PD severity having strong influence on prevalence and risk estimates. The findings of this review contribute to understanding of PD prognosis and implicate the necessity for improved quality and reporting of observational studies in the field. The clinical implication of these findings is that dental and medical professionals should be made aware of the possible association and make appropriate treatment/prevention arrangements to care. Given the strain on dental appointments following the COVID-19 pandemic, self-managed oral hygiene should also be encouraged to prevent progression to severe PD.

## Conclusion

The findings of this systematic review reveal that PD is more strongly associated with cognitive decline than dementia/AD. Meta-regression showed that some study factors may influence prevalence and risk estimates of cognitive disorders. More homologous observational evidence with clear adjustments for confounding and selection biases is required to determine the true direction of these associations. Specifically, future studies should utilise bias-reducing selection methods such as inverse probability weighting and random sampling of large and representative study populations with validated PD assessment tools to reduce the heterogeneity that is reflected in the current literature.

## Supplementary Material

aa-22-1117-File002_afad015

## Data Availability

All data generated or analysed during this study are included in this published article [and its supplementary information files].
