# Supplementary material for: The impact of study factors in the association of periodontal disease and cognitive disorders: systematic review and meta-analysis
Source: Age Ageing. 2023 Feb 14;52(2):afad015. doi: 10.1093/ageing/afad015 (PMC10789237; doi:10.1093/ageing/afad015)
Supplement: aa-22-1117-File002_afad015 [file aa-22-1117-file002_afad015.docx]

**Supplementary material:** The impact of study factors in the association of periodontal disease and cognitive disorders: systematic review and meta-analysis

**Note S1**

**Table S1.**  Summary of ROBINS-I assessment tool.

**Figure S1.** PRISMA study selection flow chart.

**Figure S2.** Funnel plots depicting publication bias in a) cognitive decline and b) dementia/AD cross-sectional studies.

**Figure S3.** Funnel plots depicting publication bias in a) cognitive decline and b) dementia/AD longitudinal studies.

**Figure S4.** Forest plot showing results from random effect meta-analysis for the prevalence of cognitive disorders by periodontal disease severity.

**Figure S5.** Forest plot showing results from random effect meta-analysis for the prevalence of cognitive disorders by region.

**Figure S6.** Forest plot showing results from random effect meta-analysis for the prevalence of cognitive disorders by dichotomised average age of population (median split 76.1 years).

**Figure S7.** Forest plot showing results from random effect meta-analysis for the incident risk cognitive disorders by periodontal disease severity.

**Figure S8.** Forest plot showing results from random effect meta-analysis for the incident risk of cognitive disorders by dichotomised average age of population at baseline (median split 70.5 years).

**Figure S9.** Forest plot showing results from random effect meta-analysis for the incident risk of cognitive disorders by study region.

**Figure S10.** Forest plot showing results from random effect meta-analysis for the incident risk of dementia in studies that treated periodontal disease during follow up.

**Figure S11.** Forest plot showing results from random effect meta-analysis for the prevalence of cognitive disorders, including studies with less than 30 participants in exposed/unexposed.

**Figure S12.** Forest plot showing results from random effect meta-analysis for the incident risk of cognitive disorders, including studies with less than 30 participants in exposed/unexposed.

**Note S1**

*Data management*

Studies retrieved from searches were imported into a citation manager and screened for duplicates using an automated system. One author screened for title and abstract for eligibility with validation by a second author. Full text articles were examined against the eligibility criteria before data extraction and quality assessment by two authors. Disputes were discussed at length and resolution made by a third author. A data extraction form was developed prior to the database search in order to identify key study information including population demographics, data source, exclusion criteria, follow up period, type of outcome measure Average age was extracted (mean or median); where age was reported in bands, we extracted the middle value of the mode age band. Data extraction was monitored by a second author and queries were discussed at length to ensure adherence to the protocol.

*Eligibility criteria*

The minimum follow-up period for longitudinal studies was 1 year following periodontal disease diagnosis and populations to ensure accurate dementia incidence calculation, rather than prevalence of an undiagnosed condition that may have preceded periodontal disease. Clinical periodontal disease classifications comprised clinical examination or identification of appropriate periodontal disease codes, such as (ICD or Read codes) within electronic health records/insurance database. Questionnaire or interview responses were denoted as self-reported periodontal disease. Evaluation of dental hygiene, presence of dental caries, cysts, lesions and other acute conditions such as gingivitis, peri-implantitis and odontogenic infection were not accepted as case definitions for periodontal disease as they may not be directly attributed to periodontal disease (British Society of Periodontology 2019).

Exclusion from the review was elicited if the study fell under one of the following criteria:

- Animal studies.
- Protocols, abstracts, reviews or conference proceedings.
- Edentulous population or lack of validated or clearly defined diagnosis of periodontal disease.

**Table S1.**  Summary of ROBINS-I assessment tool.

| **Author** | **Confounding** | **Selection** | **Exposure classification** | **Exposure deviations** | **Missing data** | **Outcome measure** | **Reporting bias** | **Overall** |
| --- | --- | --- | --- | --- | --- | --- | --- | --- |
| ***Cross-sectional studies*** | |  |  |  |  |  |  |  |
| Adulhade Ganem 2019 | SERIOUS | SERIOUS | LOW | LOW | SERIOUS | CRITICAL | CRITICAL | CRITICAL |
| Alfotawi 2020 | SERIOUS | SERIOUS | LOW | LOW | MODERATE | SERIOUS | MODERATE | SERIOUS |
| Barbe 2020 | SERIOUS | SERIOUS | LOW | SERIOUS | MODERATE | SERIOUS | LOW | SERIOUS |
| Chu 2015 | SERIOUS | SERIOUS | LOW | LOW | MODERATE | MODERATE | LOW | SERIOUS |
| Gao 2020 | SERIOUS | MODERATE | LOW | LOW | MODERATE | SERIOUS | LOW | SERIOUS |
| Jockusch 2021 | SERIOUS | MODERATE | LOW | LOW | SERIOUS | SERIOUS | LOW | SERIOUS |
| Kato 2019 | SERIOUS | MODERATE | LOW | LOW | SERIOUS | SERIOUS | SERIOUS | SERIOUS |
| Kim 2021 | SERIOUS | SERIOUS | LOW | LOW | MODERATE | LOW | SERIOUS | SERIOUS |
| Laugisch 2021 | SERIOUS | SERIOUS | LOW | LOW | MODERATE | LOW | MODERATE | SERIOUS |
| Mizutani 2021 | MODERATE | SERIOUS | LOW | LOW | LOW | SERIOUS | LOW | SERIOUS |
| Nilsson 2014 | SERIOUS | MODERATE | LOW | LOW | MODERATE | LOW | LOW | SERIOUS |
| Nilsson 2018 | SERIOUS | MODERATE | LOW | LOW | MODERATE | LOW | SERIOUS | SERIOUS |
| Okamoto 2010 | SERIOUS | SERIOUS | LOW | LOW | MODERATE | LOW | LOW | SERIOUS |
| Peres 2015 | MODERATE | MODERATE | MODERATE | MODERATE | LOW | LOW | LOW | MODERATE |
| Popovac 2021 | SERIOUS | SERIOUS | LOW | LOW | MODERATE | SERIOUS | SERIOUS | SERIOUS |
| Saito 2021 | MODERATE | MODERATE | LOW | LOW | MODERATE | LOW | LOW | MODERATE |
| Sharma 2021 | MODERATE | SERIOUS | LOW | SERIOUS | MODERATE | LOW | LOW | SERIOUS |
| Shin 2016 | MODERATE | MODERATE | LOW | SERIOUS | MODERATE | LOW | LOW | SERIOUS |
| Tiisanjoa 2019 | MODERATE | MODERATE | LOW | MODERATE | MODERATE | LOW | LOW | MODERATE |
| Tsuneishi 2021 | SERIOUS | LOW | LOW | SERIOUS | MODERATE | LOW | SERIOUS | SERIOUS |
| Winning 2022 | MODERATE | LOW | LOW | LOW | MODERATE | LOW | LOW | MODERATE |
| ***Longitudinal studies*** | |  |  |  |  |  |  |  |
| Adam 2022 | LOW | SERIOUS | LOW | LOW | MODERATE | MODERATE | MODERATE | SERIOUS |
| Arrive 2012 | MODERATE | SERIOUS | LOW | LOW | SERIOUS | LOW | CRITICAL | CRITICAL |
| Batty 2013 | MODERATE | SERIOUS | MODERATE | MODERATE | SERIOUS | LOW | SERIOUS | SERIOUS |
| Chen 2017 | SERIOUS | MODERATE | LOW | LOW | SERIOUS | LOW | MODERATE | SERIOUS |
| Choi 2019 | SERIOUS | MODERATE | LOW | LOW | SERIOUS | MODERATE | SERIOUS | SERIOUS |
| Demmer 2020 | LOW | MODERATE | LOW | LOW | SERIOUS | LOW | LOW | MODERATE |
| Govindan 2021 | MODERATE | SERIOUS | LOW | MODERATE | SERIOUS | LOW | SERIOUS | SERIOUS |
| Hatta 2018 | MODERATE | SERIOUS | LOW | MODERATE | MODERATE | LOW | SERIOUS | SERIOUS |
| Holmer 2022 | SERIOUS | MODERATE | LOW | SERIOUS | SERIOUS | SERIOUS | CRITICAL | CRITICAL |
| Iwasaki 2019 | LOW | MODERATE | LOW | LOW | MODERATE | LOW | LOW | MODERATE |
| Kim 2020 | MODERATE | MODERATE | LOW | MODERATE | SERIOUS | LOW | SERIOUS | SERIOUS |
| Kiuchi 2021 | LOW | SERIOUS | MODERATE | MODERATE | LOW | MODERATE | LOW | SERIOUS |
| Lee 2017a | SERIOUS | SERIOUS | LOW | MODERATE | SERIOUS | LOW | MODERATE | SERIOUS |
| Lee 2017b | SERIOUS | MODERATE | LOW | LOW | SERIOUS | LOW | MODERATE | SERIOUS |
| Lee 2020 | SERIOUS | MODERATE | LOW | LOW | SERIOUS | LOW | SERIOUS | SERIOUS |
| Malone 2021 | MODERATE | SERIOUS | LOW | SERIOUS | SERIOUS | LOW | LOW | SERIOUS |
| Nilsson 2018 | MODERATE | MODERATE | LOW | LOW | SERIOUS | LOW | MODERATE | SERIOUS |
| Okamoto 2018 | MODERATE | SERIOUS | LOW | MODERATE | MODERATE | LOW | CRITICAL | CRITICAL |
| Paganini-Hill 2012 | LOW | SERIOUS | MODERATE | MODERATE | SERIOUS | MODERATE | SERIOUS | SERIOUS |
| Saito 2018 | MODERATE | SERIOUS | MODERATE | LOW | MODERATE | LOW | MODERATE | SERIOUS |
| Stein 2007 | LOW | MODERATE | MODERATE | MODERATE | SERIOUS | LOW | MODERATE | SERIOUS |
| Stewart 2015 | MODERATE | SERIOUS | MODERATE | MODERATE | MODERATE | LOW | MODERATE | SERIOUS |
| Takeuchi 2017 | SERIOUS | SERIOUS | LOW | MODERATE | SERIOUS | SERIOUS | MODERATE | SERIOUS |
| Tzeng 2016 | SERIOUS | MODERATE | LOW | LOW | SERIOUS | LOW | MODERATE | SERIOUS |
| Xu 2021 | MODERATE | SERIOUS | MODERATE | MODERATE | SERIOUS | LOW | LOW | SERIOUS |
| Yamamoto 2012 | MODERATE | SERIOUS | MODERATE | MODERATE | MODERATE | LOW | LOW | SERIOUS |
| Yang 2022 | MODERATE | SERIOUS | MODERATE | MODERATE | LOW | LOW | LOW | SERIOUS |
| Yoo 2019 | SERIOUS | MODERATE | LOW | MODERATE | MODERATE | LOW | LOW | SERIOUS |

**Figure S1.** PRISMA study selection flow chart.


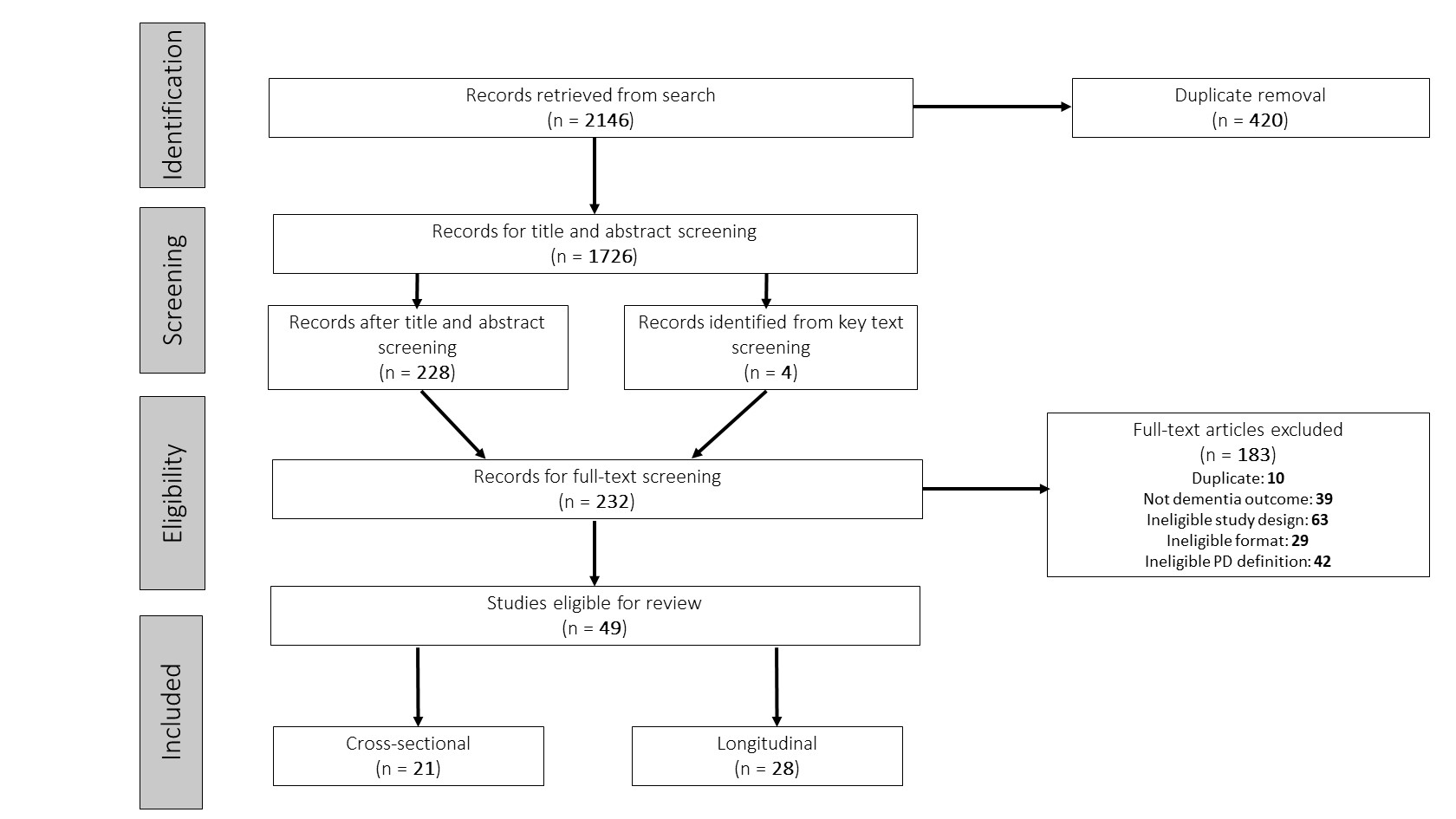


**Key:** periodontal disease (PD).

**Figure S2.** Funnel plots depicting publication bias in a) cognitive decline and b) dementia/AD cross-sectional studies.

**a)**


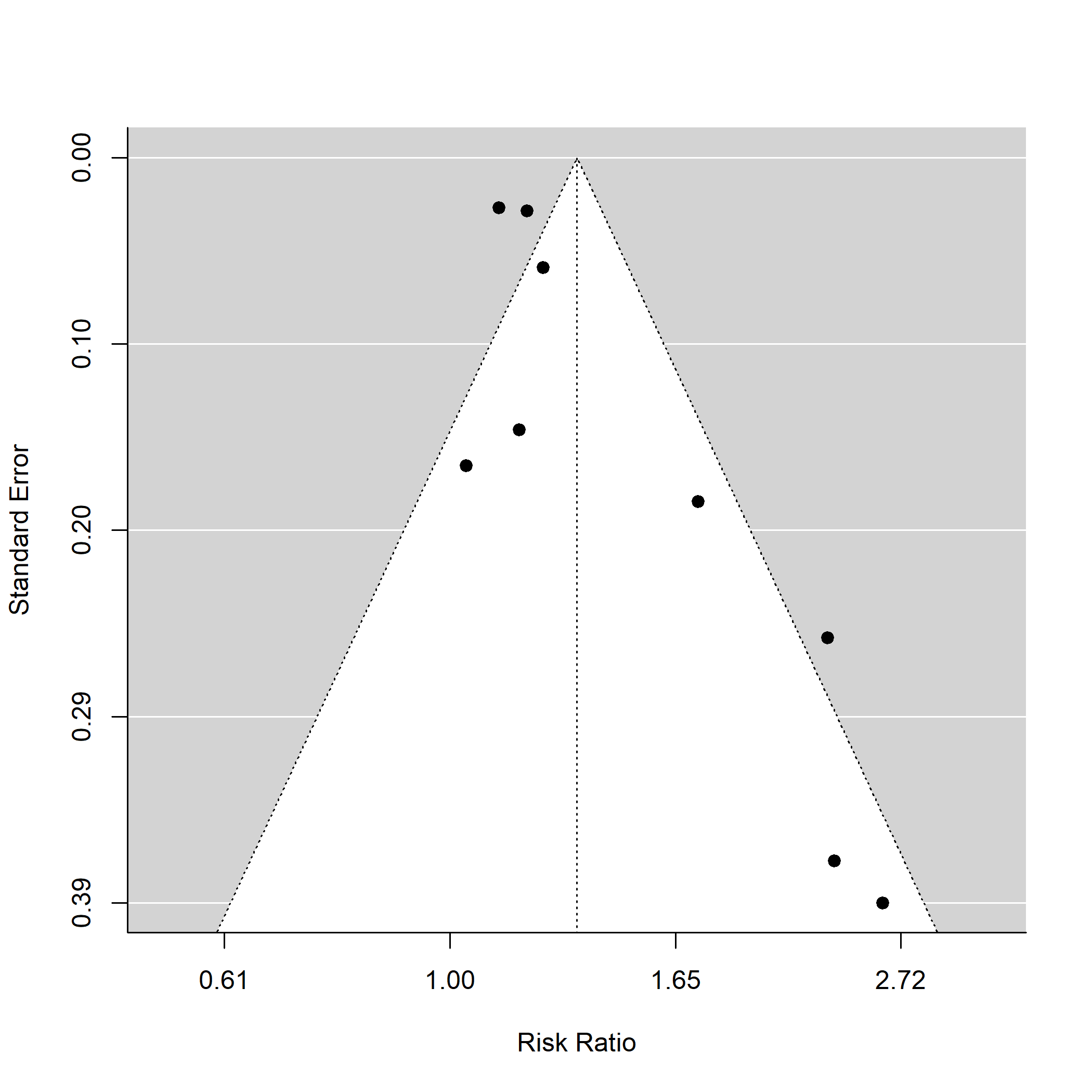


*Eggers test for funnel plot asymmetry: z = 3.5704, p < 0.05.*

**b)** *Eggers test for funnel plot asymmetry: z = 1.1224, p = 0.2617.*


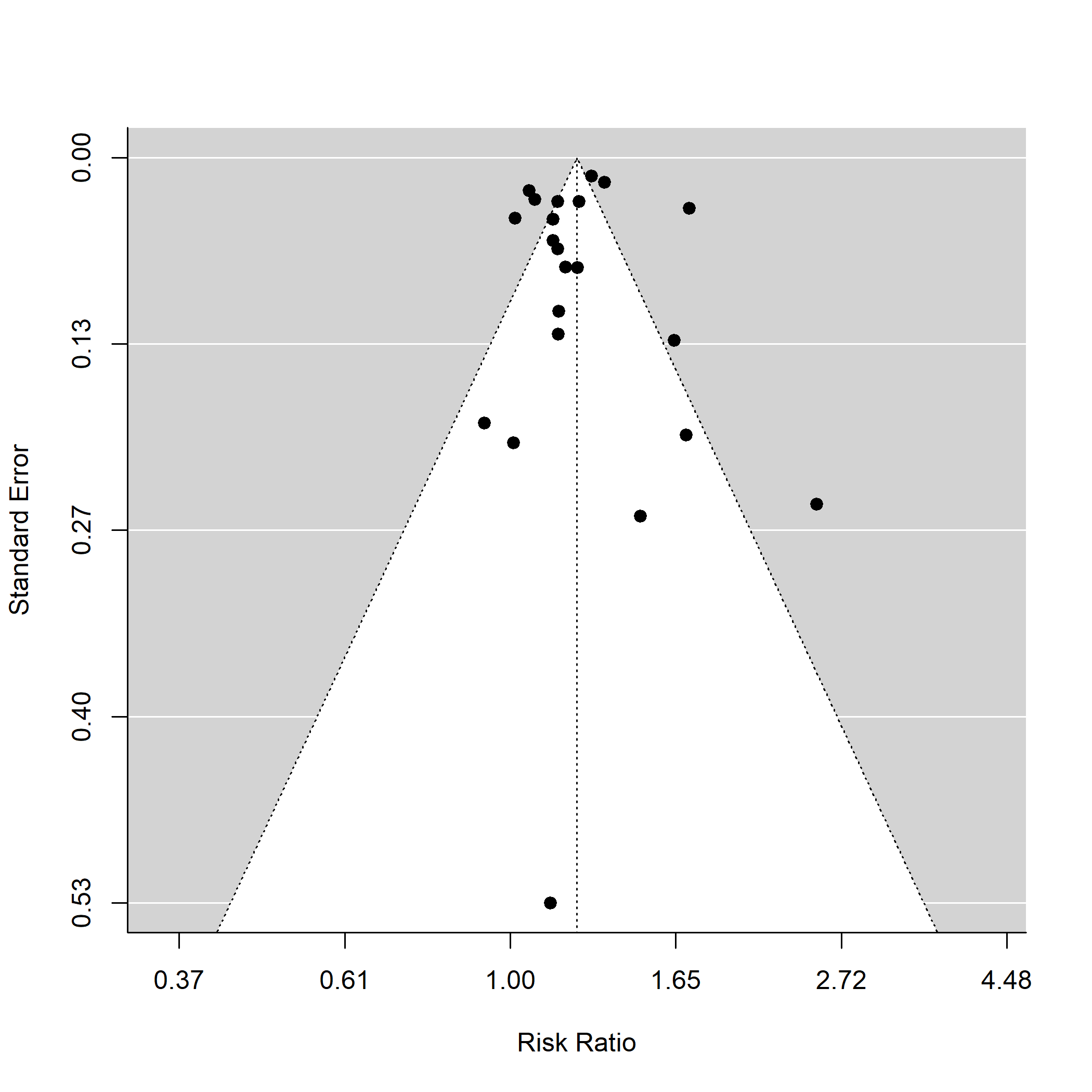


**Figure S3.** Funnel plots depicting publication bias in a) cognitive decline and b) dementia/AD longitudinal studies.

**a)**

*Eggers test for funnel plot asymmetry: z = 3.5704, p < 0.05*


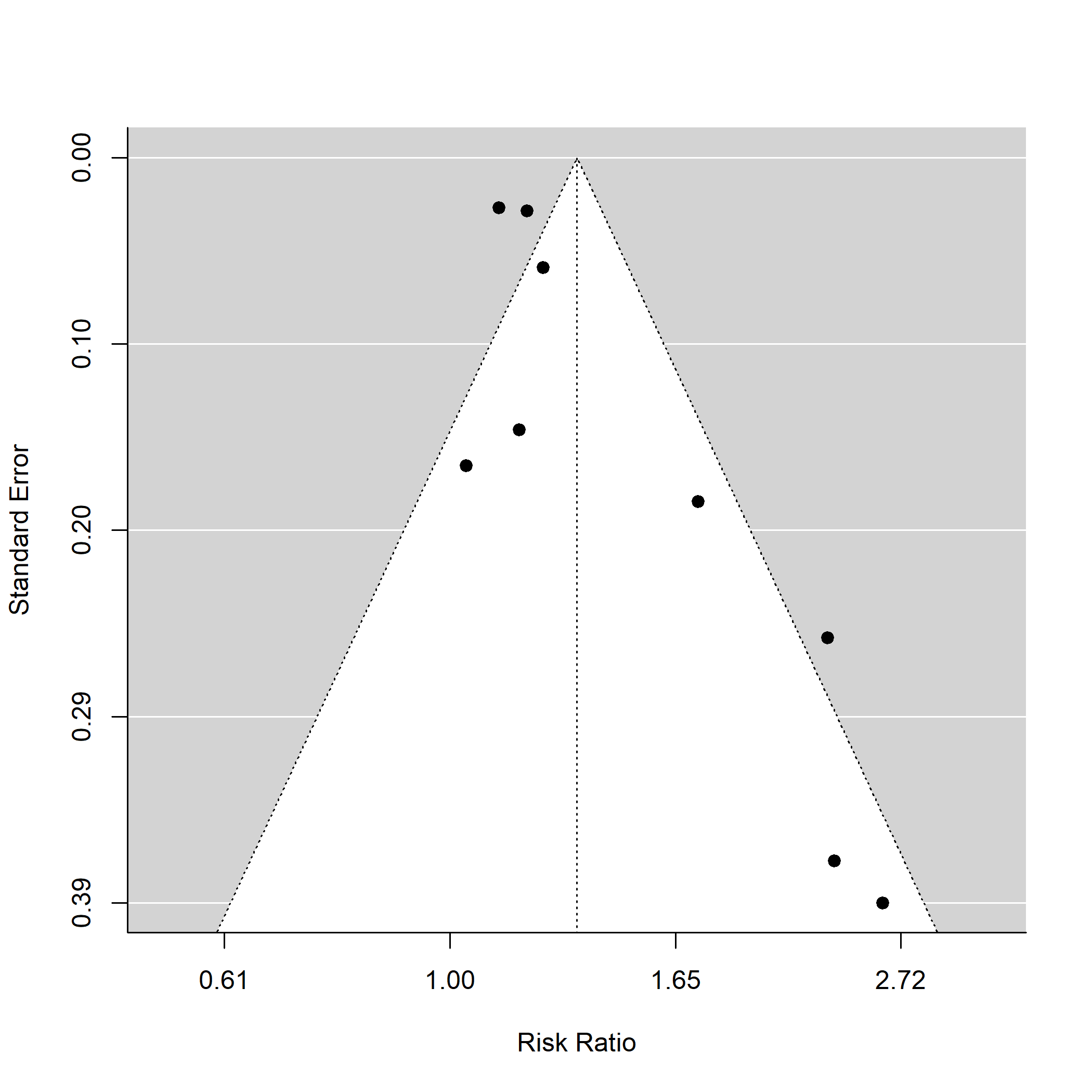


**b)**


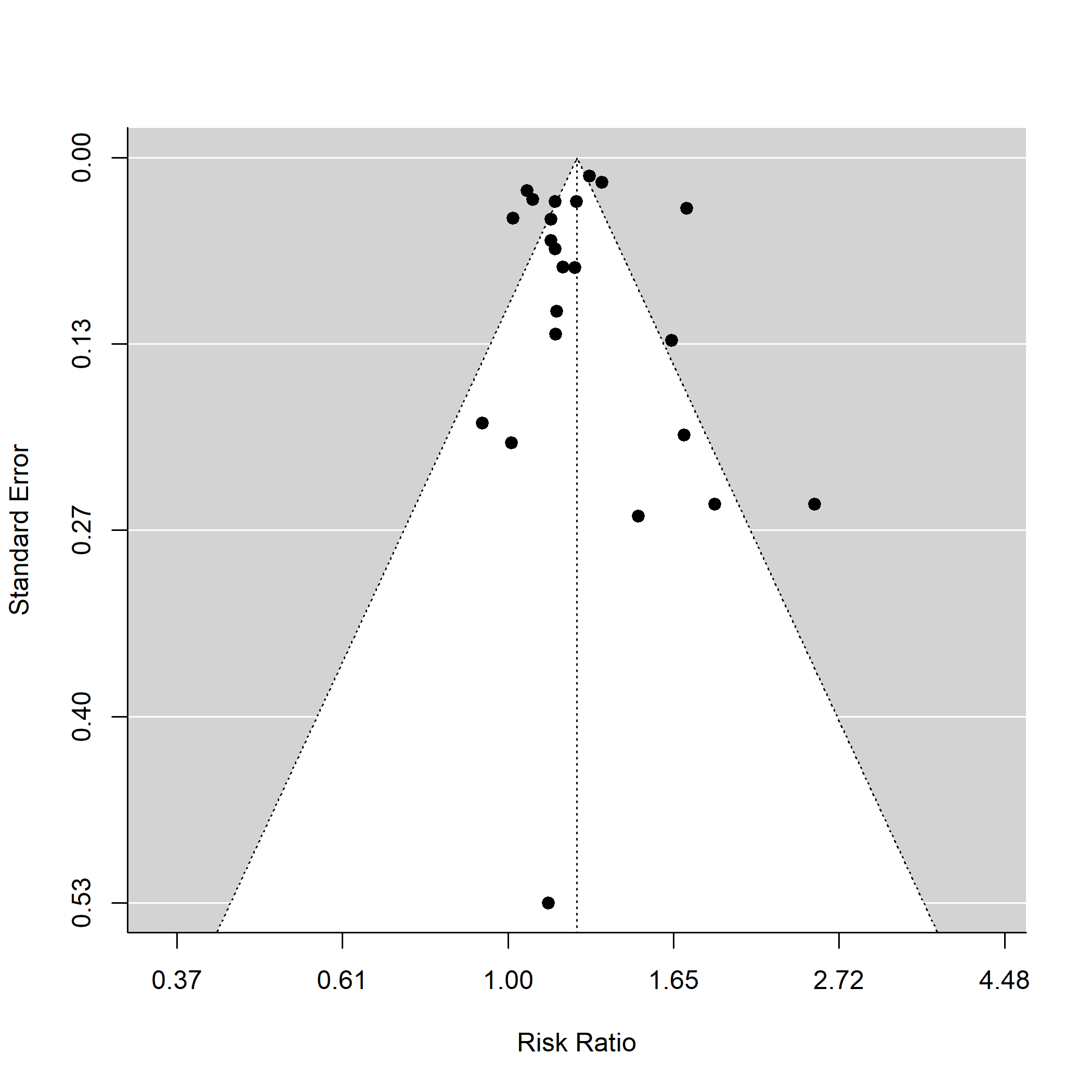


*Eggers test for funnel plot asymmetry z = 1.5369, p = 0.1243*

**Figure S4.** Forest plot showing results from random effect meta-analysis for the prevalence of cognitive disorders by periodontal disease severity.


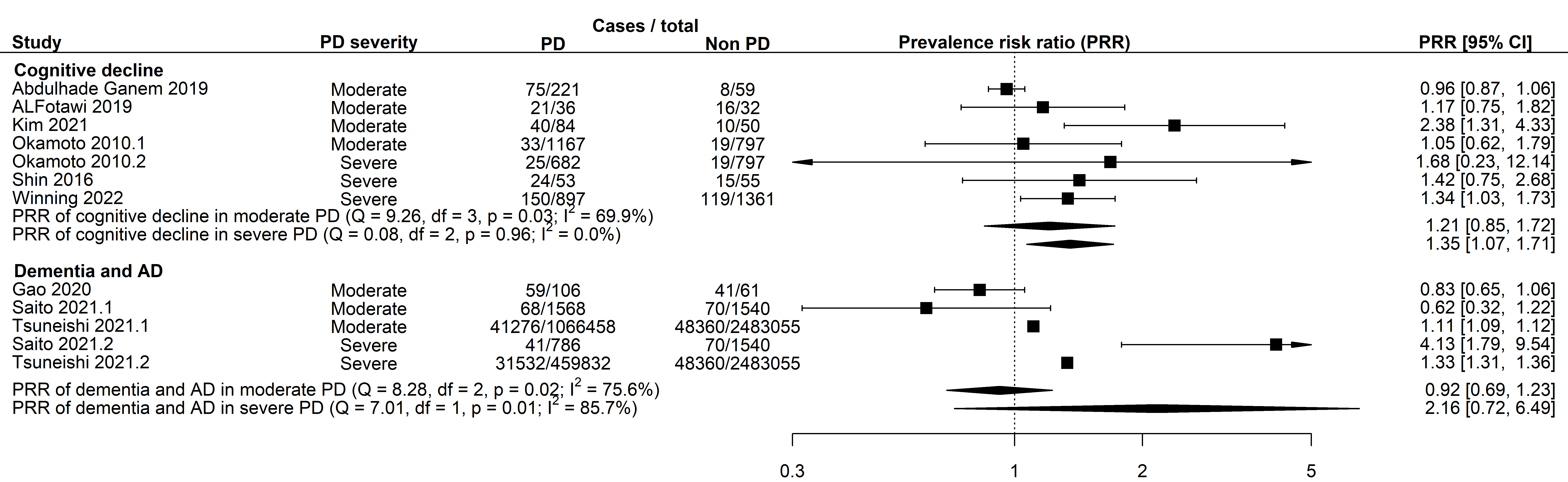


**Key:** Alzheimer’s disease (AD), degrees of freedom (df), periodontal disease (PD), prevalence odds ratio (POR).

**Figure S5.** Forest plot showing results from random effect meta-analysis for the prevalence of cognitive disorders by region.


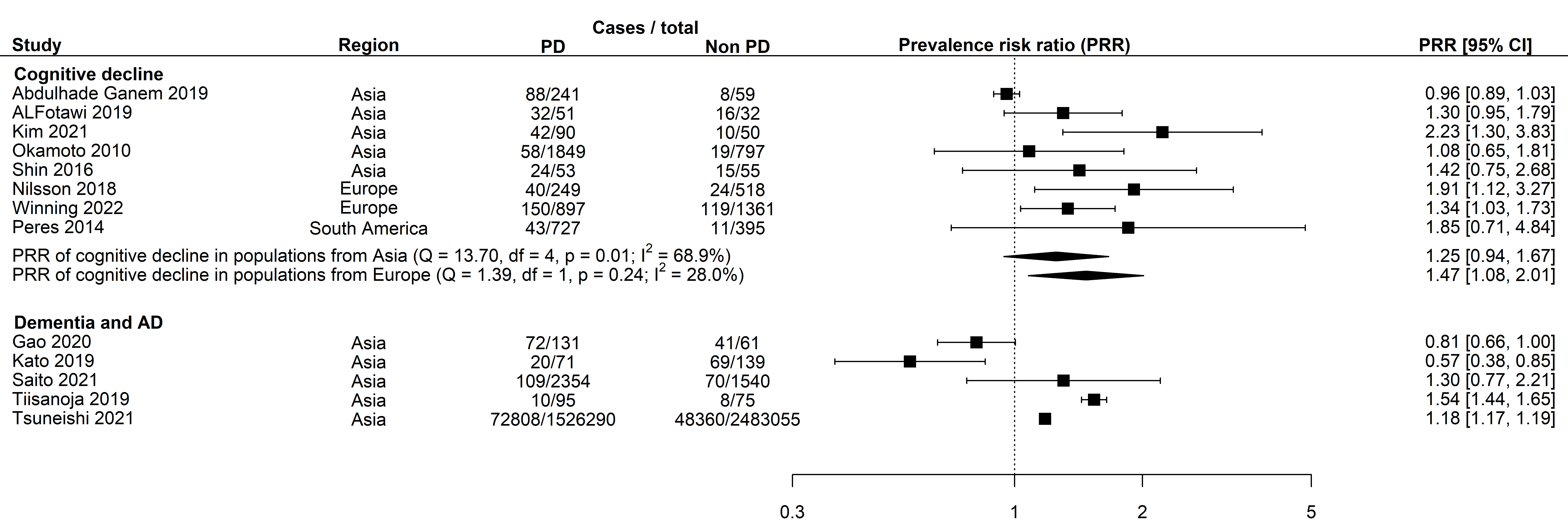


**Key:** Alzheimer’s disease (AD), degrees of freedom (df), periodontal disease (PD), prevalence odds ratio (POR).

**Figure S6.** Forest plot showing results from random effect meta-analysis for the prevalence of cognitive disorders by dichotomised average age of population (median split 76.1 years).


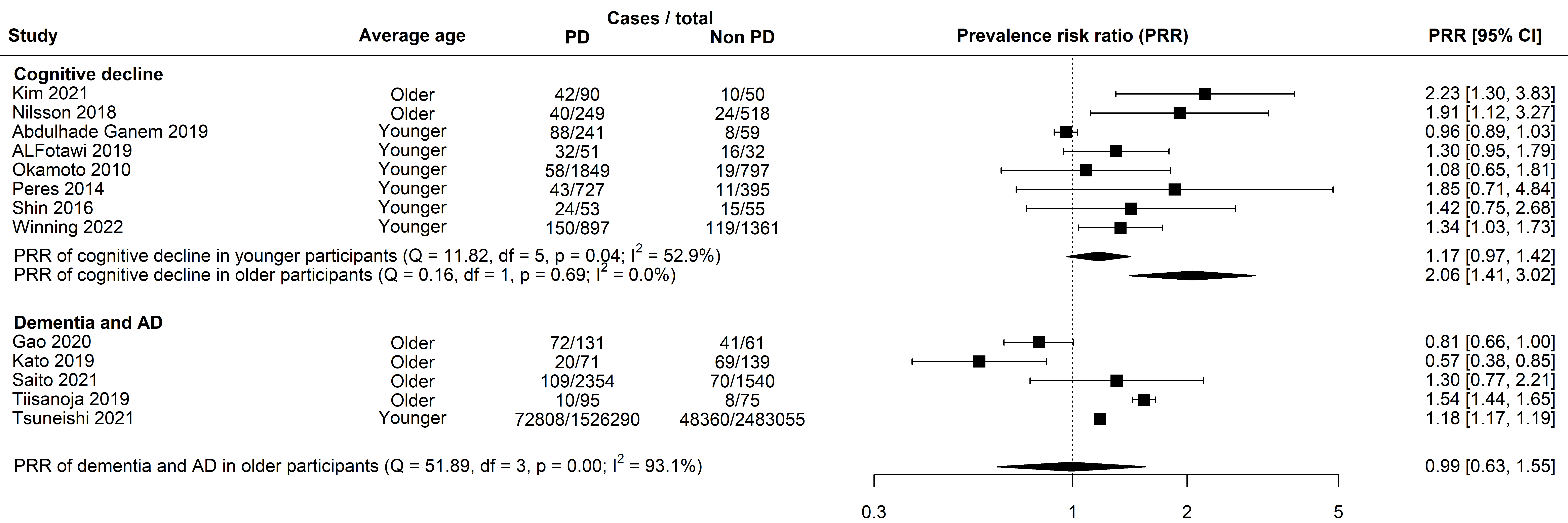


**Key:** Alzheimer’s disease (AD), degrees of freedom (df), periodontal disease (PD), prevalence odds ratio (POR).

**Figure S7.** Forest plot showing results from random effect meta-analysis for the incident risk cognitive disorders by periodontal disease severity.


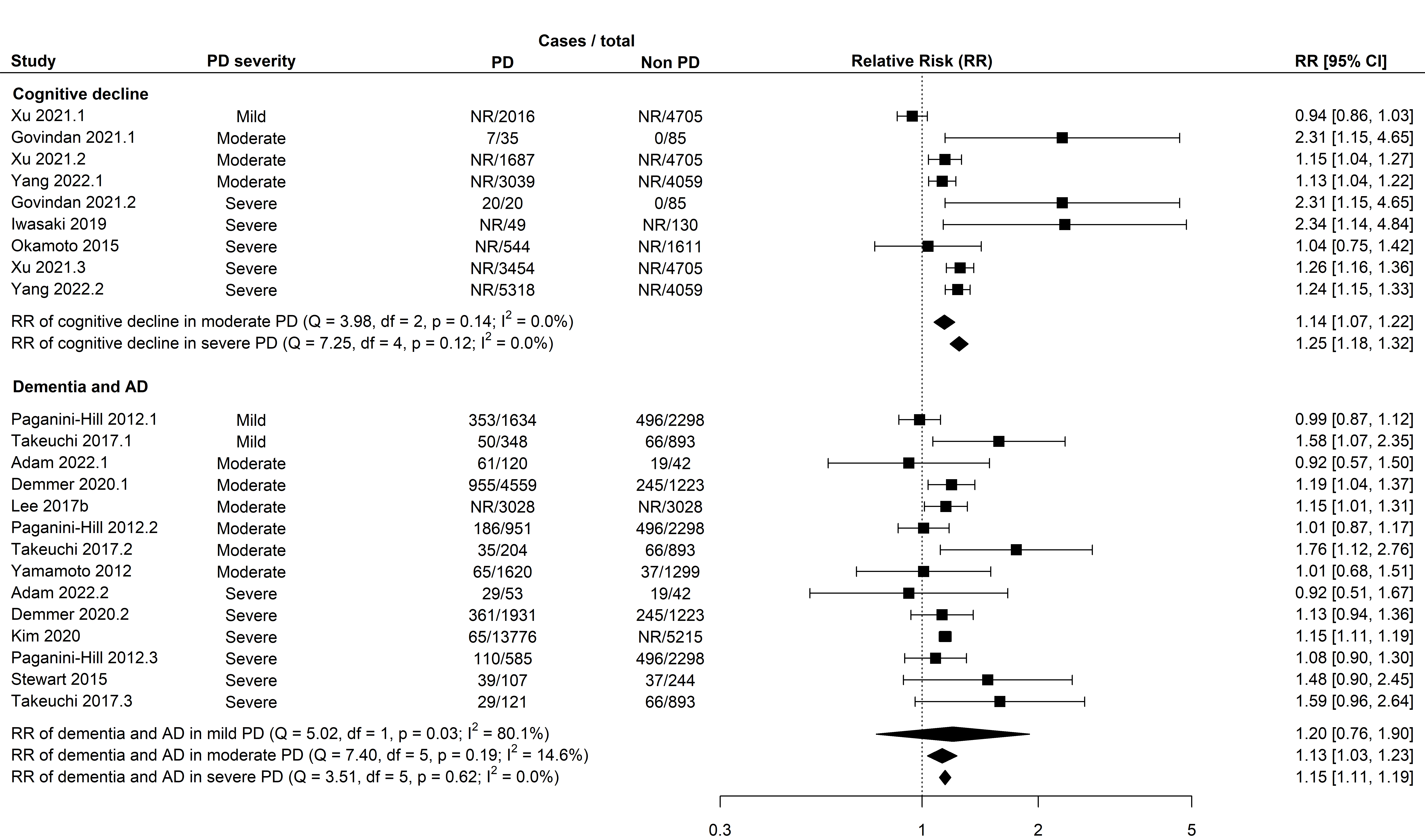


**Key:** Alzheimer’s disease (AD), degrees of freedom (df), case numbers not reported (NR), periodontal disease (PD), relative risk (RR).

**Figure S8.** Forest plot showing results from random effect meta-analysis for the incident risk of cognitive disorders by dichotomised average age of population at baseline (median split 70.5 years).


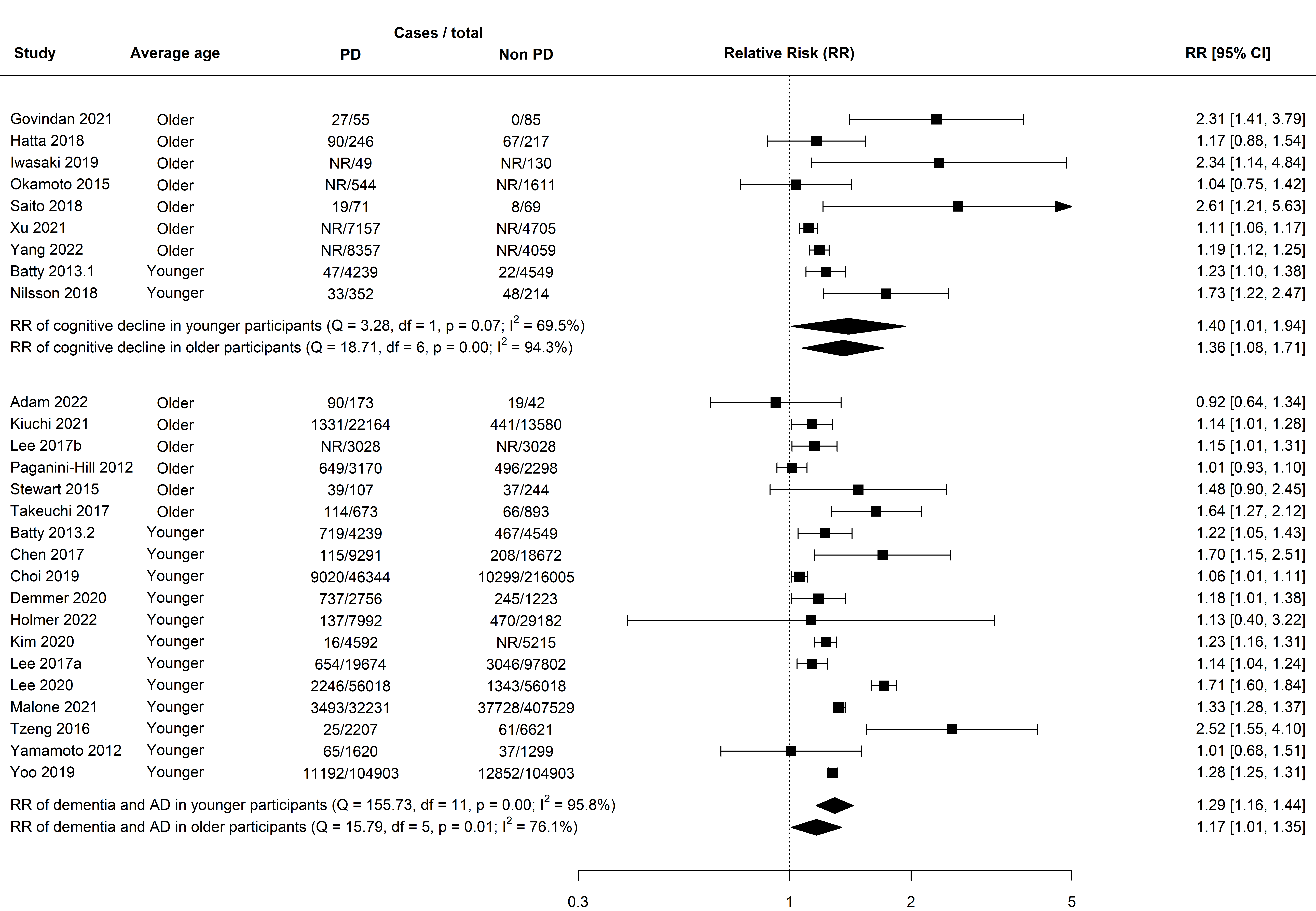


**Key:** Alzheimer’s disease (AD), degrees of freedom (df), case numbers not reported (NR), periodontal disease (PD), relative risk (RR).

**Figure S9.** Forest plot showing results from random effect meta-analysis for the incident risk of cognitive disorders by study region.


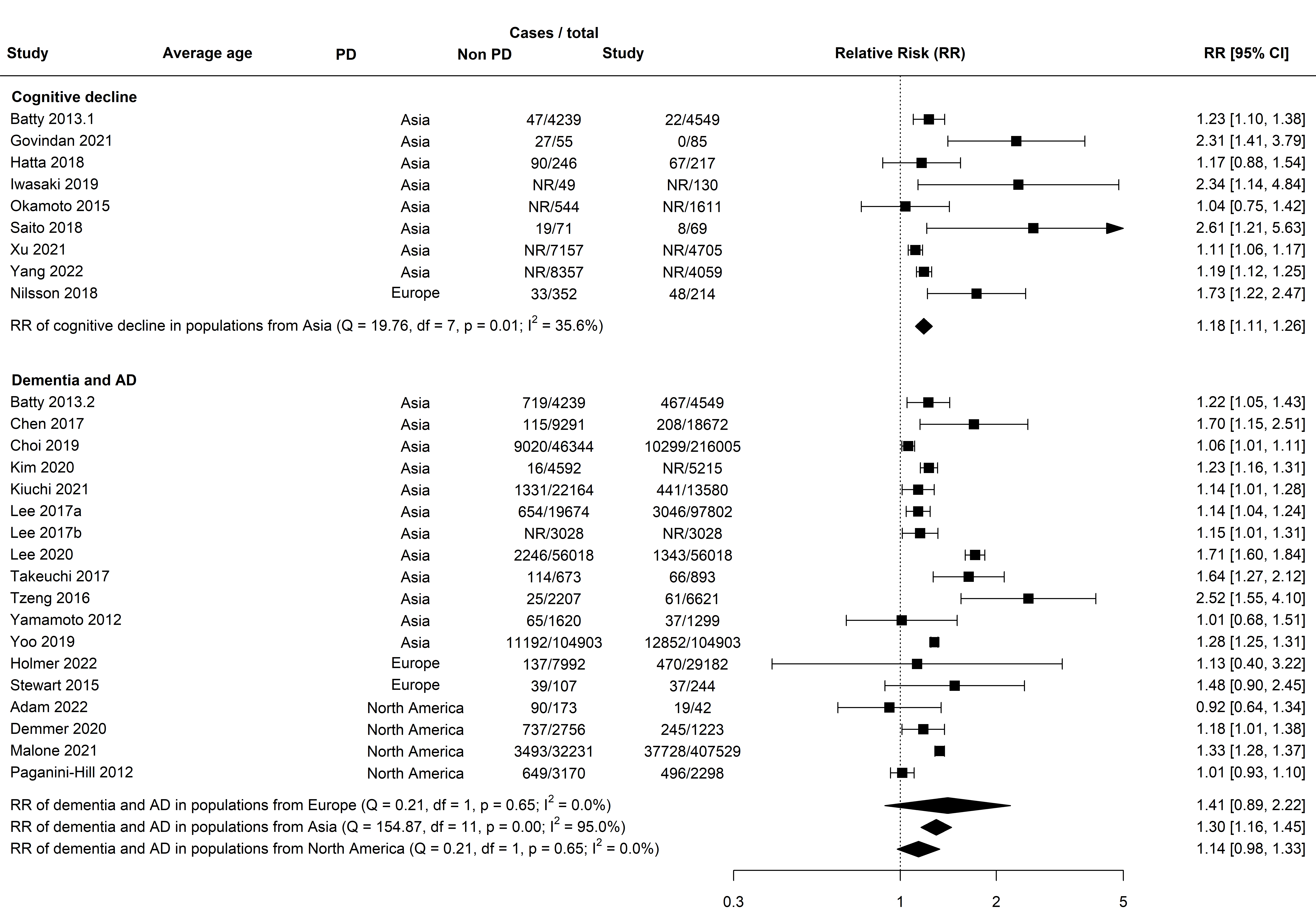


**Key:** Alzheimer’s disease (AD), degrees of freedom (df), case numbers not reported (NR), periodontal disease (PD), relative risk (RR)

**Figure S10.** Forest plot showing results from random effect meta-analysis for the incident risk of dementia in studies that treated periodontal disease during follow up.


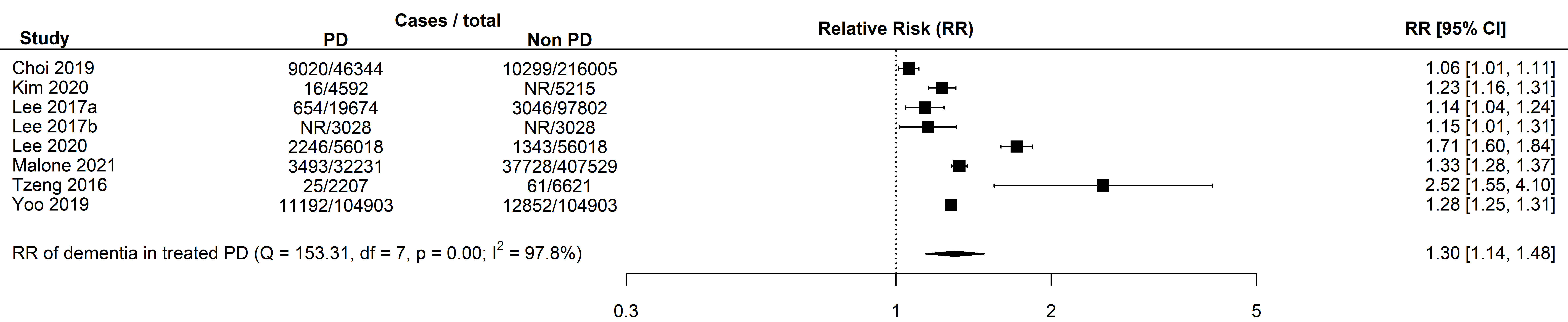


**Key:** Alzheimer’s disease (AD), degrees of freedom (df), case numbers not reported (NR), periodontal disease (PD), relative risk (RR).

**Figure S11.** Forest plot showing results from random effect meta-analysis for the prevalence of cognitive disorders, including studies with less than 30 participants in exposed/unexposed.


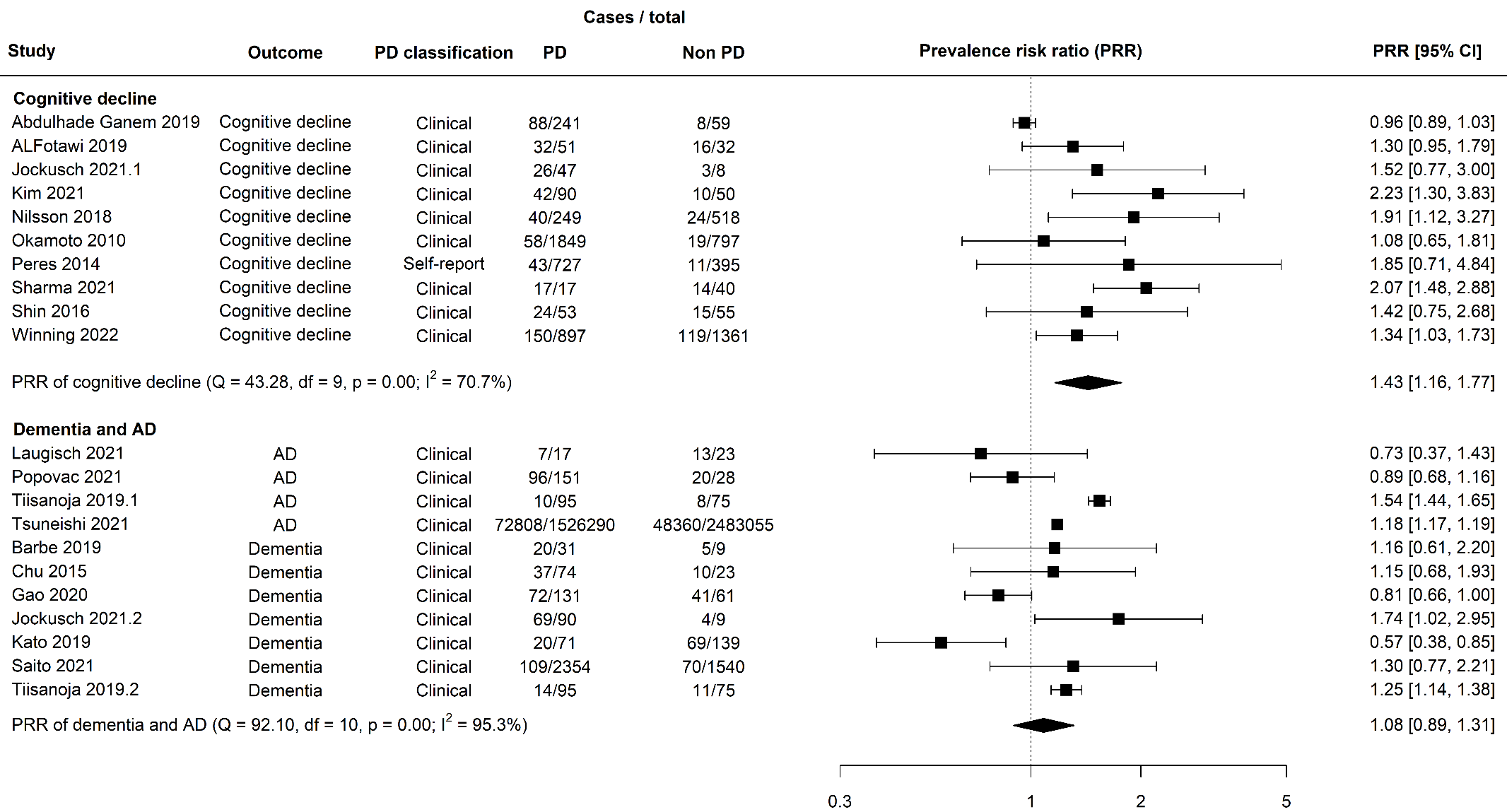


**Key:** Alzheimer’s disease (AD), degrees of freedom (df), periodontal disease (PD), prevalence risk ratio (PRR).

**Figure S12.** Forest plot showing results from random effect meta-analysis for the incident risk of cognitive disorders, including studies with less than 30 participants in exposed/unexposed.


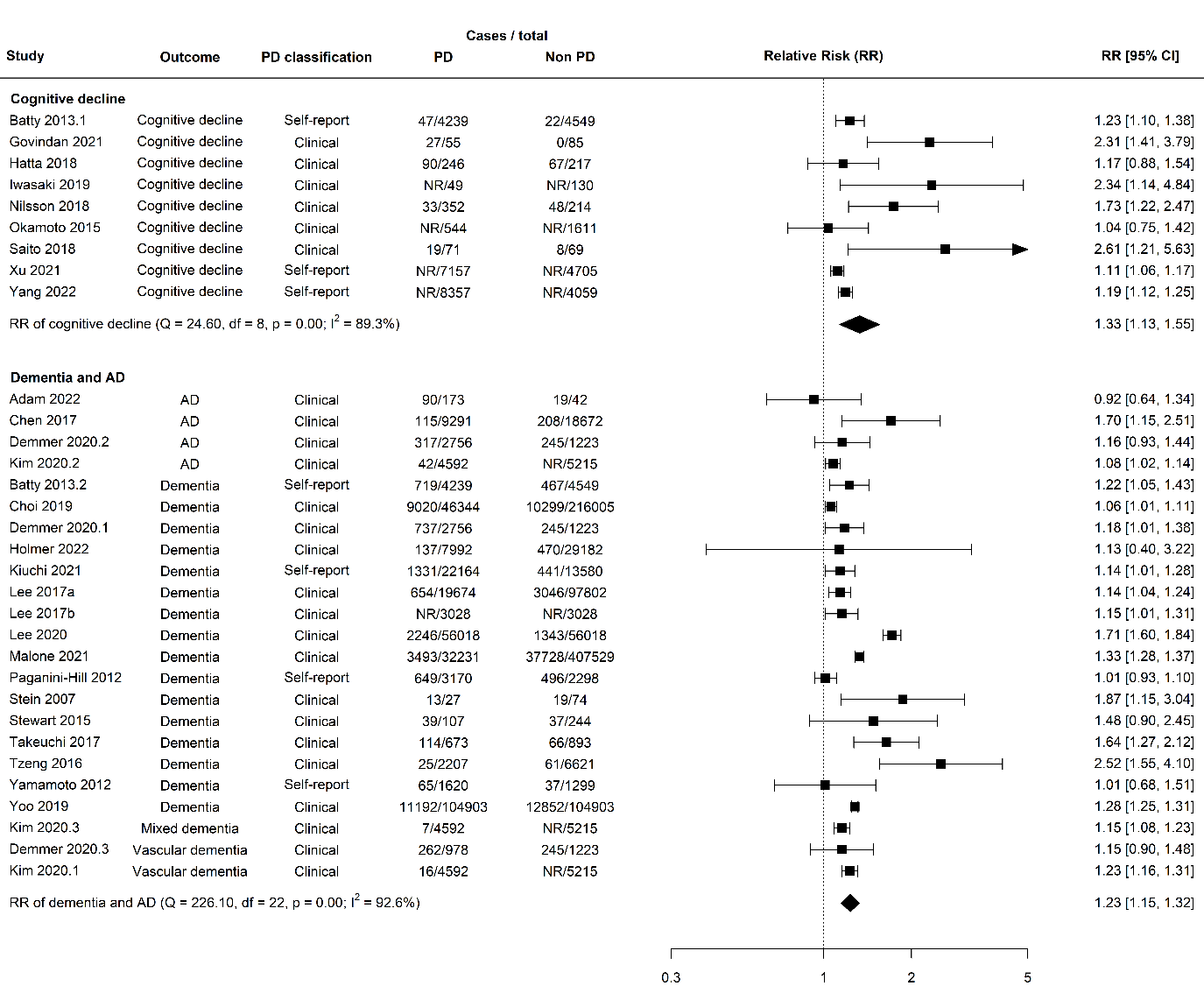


**Key:** Alzheimer’s disease (AD), degrees of freedom (df), case numbers not reported (NR), periodontal disease (PD), relative risk (RR)
